# Characterization of rifampicin-resistant *Mycobacterium tuberculosis* in Khyber Pakhtunkhwa, Pakistan

**DOI:** 10.1038/s41598-021-93501-4

**Published:** 2021-07-09

**Authors:** Anwar Sheed Khan, Jody E. Phelan, Muhammad Tahir Khan, Sajid Ali, Muhammad Qasim, Gary Napier, Susana Campino, Sajjad Ahmad, Otavio Cabral-Marques, Shulin Zhang, Hazir Rahman, Dong-Qing Wei, Taane G. Clark, Taj Ali Khan

**Affiliations:** 1 Department of Microbiology, Kohat University of Science and Technology, Kohat, Pakistan; 2 Provincial Tuberculosis Reference, Laboratory Hayatabad Medical Complex, Peshawar, Pakistan; 3 Faculty of Infectious and Tropical Diseases, London School of Hygiene & Tropical Medicine, London, UK; 4 Institute of Molecular Biology and Biotechnology (IMBB), The University of Lahore. KM, Defense Road, Lahore, 58810 Pakistan; 5 Institute of Basic Medical Science, Khyber Medical University, Peshawar, KP Pakistan; 6 Department of Immunology, Institute of Biomedical Sciences, University of Sao Paulo, São Paulo, SP Brazil; 7 Department of Clinical and Toxicological Analyses, School of Pharmaceutical Sciences, University of São Paulo, São Paulo, SP Brazil; 8 Network of Immunity in Infection, Malignancy, and Autoimmunity (NIIMA), Universal Scientific Education and Research Network (USERN), São Paulo, Brazil; 9 Department of Microbiology and Immunology, Shanghai Jiao Tong University School of Medicine, Shanghai, China; 10 Department of Microbiology, Abdul Wali Khan University, Mardan, Pakistan; 11 State Key Laboratory of Microbial Metabolism, School of Life Sciences and Biotechnology, and Joint Laboratory of International Cooperation in Metabolic and Developmental Sciences, Ministry of Education, Shanghai Jiao Tong University, 800 Dongchuan Road, Minhang District, Shanghai, 200240 China; 12 Faculty of Epidemiology and Population Health, London School of Hygiene & Tropical Medicine, London, UK

**Keywords:** Genomics, Microbial genetics, Bacterial genetics

## Abstract

Tuberculosis (TB), caused by *Mycobacterium tuberculosis,* is endemic in Pakistan. Resistance to both firstline rifampicin and isoniazid drugs (multidrug-resistant TB; MDR-TB) is hampering disease control. Rifampicin resistance is attributed to *rpoB* gene mutations, but *rpoA* and *rpoC l*oci may also be involved. To characterise underlying rifampicin resistance mutations in the TB endemic province of Khyber Pakhtunkhwa, we sequenced 51 *M. tuberculosis* isolates collected between 2016 and 2019; predominantly, MDR-TB (n = 44; 86.3%) and lineage 3 (n = 30, 58.8%) strains. We found that known mutations in *rpoB* (e.g. S405L), *katG* (e.g. S315T), or *inhA* promoter loci explain the MDR-TB. There were 24 unique mutations in *rpoA, rpoB,* and *rpoC* genes, including four previously unreported. Five instances of within-host resistance diversity were observed, where two were a mixture of MDR-TB strains containing mutations in *rpoB, katG,* and the *inhA* promoter region, as well as compensatory mutations in *rpoC.* Heteroresistance was observed in two isolates with a single lineage. Such complexity may reflect the high transmission nature of the Khyber Pakhtunkhwa setting. Our study reinforces the need to apply sequencing approaches to capture the full-extent of MDR-TB genetic diversity, to understand transmission, and to inform TB control activities in the highly endemic setting of Pakistan.

## Introduction

Tuberculosis (TB), caused by *Mycobacterium tuberculosis* (MTB), affects ~ 10 million people annually^1^. Despite a recent reduction in TB incidence, increasing drug resistance is making infection control difficult^1^. New diagnostic tools and treatments are needed in developing countries to inform clinical and disease control activities^2^. The recommended combination of anti-TB drugs involves a 6-month standard regimen including first-line rifampicin, isoniazid, ethambutol, and pyrazinamide^1^. The detection of *M. tuberculosis* resistant to first-line rifampicin (RR-TB) and isoniazid, together called multidrug resistance (MDR-TB), is especially crucial in Pakistan^3^. The country is in the top eight for RR-TB and MDR-TB burden, with an estimated 6100 of its > 330,000 TB cases in 2019 presenting with RR-TB or greater resistance^4^.

Khyber Pakhtunkhwa is the third largest province of Pakistan, with an area of 74,521 km^2^ and a population size of approximately 30.5 million individuals. It has a history of armed conflicts, and TB control has been a neglected area of public health. Recently, a TB control program was launched at Hayatabad Medical Complex Peshawar to monitor disease incidence and perform drug susceptibility testing (DST) to assess levels of RR-TB and MDR-TB. RR-TB is mainly caused by the presence of mutations in the RNA polymerase β subunit (*rpoB*) gene. Mutations in an 81-bp core region of the *rpoB* gene, known as the RR-determining region (RRDR), account for 95% of the RR-TB detected in clinical isolates^5^. However, secondary mutations in the *rpoA* or *rpoC* genes can mitigate the initial fitness cost caused by a *rpoB* mutation^6,7^. About one-third of RR-TB involves *rpoB* mutant strains with companion *rpoC* or *rpoA* mutations^8^. Here, by sequencing fifty-one *M. tuberculosis*, we aimed to summarise the diversity and potential role of *rpoB, rpoA*, and *rpoC* mutations in the high MDR incidence setting of Khyber Pakhtunkhwa, to inform diagnostics tools for TB control and implementation. The analysis revealed some mixed MDR infections and heteroresistance, reflecting the complex dynamics of the high transmission setting in Pakistan, and reinforcing the need for robust diagnostic tools.

## Materials and methods

A total of 4822 pulmonary and extrapulmonary samples positive for *M. tuberculosis* were processed between 2016 to 2019 by the provincial Reference Laboratory for TB at Hayatabad Medical Complex Peshawar. Samples were received from all the Programmatic drug management units in the province. All samples were processed using the *N*-acetyl-l-cysteine–sodium hydroxide (NALC–NaOH) concentration method, using Lowenstein–Jensen medium (LJ) solid culture and liquid MGIT tubes containing 7H9 media^9^. All samples were subject to phenotypic drug susceptibility testing (DST), performed through the automated BACTEC MGIT 960 system (BD Diagnostic Systems, NJ, USA)^10^. The DST for resistant isolates was repeated for confirmation of drug resistance profile. Resistance to isoniazid, rifampicin, and other drugs was assessed through the BACTEC MGIT 960 system, with established WHO critical concentrations^11^. As per WHO guidelines for MGIT DST, there is only a single concentration (1.0 umg/mL) for rifampicin. Fifty-one samples (4 pan-susceptible, 47 drug-resistant, including 44 MDR-TB) were selected for whole genome sequencing (WGS). The source of genomic DNA was MGIT Culture (Liquid Culture) and the same culture generation was used for the phenotypic and genotypic (WGS) characterisation. The study was approved by the Institutional Ethics Committee of KUST Kohat and Provincial Tuberculosis Reference Laboratory, Khyber Pakhtunkhwa (Ref. no.: PTP/PTRL-408/19). All study participants gave informed consent for the collection and use of their biological materials, and all methods were performed in accordance with the relevant guidelines and regulations.

Genomic DNA for the fifty-one isolates was extracted using the acetyl trimethylammonium bromide (CTAB) method^12^. The DNA underwent WGS at the London School of Hygiene and Tropical Medicine (LSHTM) using the Illumina MiSeq platform (QIAseq FX DNA Library Kit) with a 151 bp paired-end protocol (v3). The quality of the resulting raw sequence data (FASTQ files) was assessed using the FastQC tool (v0.11.8). Reads were trimmed using trimmomatic software (v0.39, LEADING:3 TRAILING:3 SLIDINGWINDOW:4:20 MINLEN:36). The filtered reads were aligned to the H37Rv genome (Accession No. AL123456.3) using Burrow-Wheeler Aligner (BWA)-mem (v0.6) software^13^. The GATK tool (HaplotypeCaller v4.1.4.1, -ERC GVCF)^14^ was used to call single nucleotide polymorphisms (SNPs) and insertions and deletions (indels), and the variants were annotated for function (e.g. non-synonymous mutations, frameshifts). The *M. tuberculosis* isolates were profiled for lineage and genotypic drug resistance using TB-Profiler software (v3.0.0), which requires a minimum of 10 reads to call variants by default^15^. Mixed infections were defined as samples with more than one lineage, predicted by TB-Profiler, and supported by > 200 biallelic genomic positions. Mutations were assigned to the strains by comparing their frequency in read coverage to the predicted frequency of the strains in each mixture based on genome-wide variants. All results were compared to a mutational database consisting of > 35 k *M. tuberculosis* spanning all lineages and drug resistance types^16^. The effects of mutations on RpoA, RpoB, and RpoC protein structures were investigated using the DynaMut server^17^. In particular, the effects on protein stability (changes in vibrational entropy, ΔΔG: kcal/mol) and flexibility (ΔΔSVib: kcal/mol-1.K-1) were assessed using models downloaded from the PDB (ID: 6c04). Negative predicted stability changes are destabilising, whilst positive changes are stabilising. Consensus genomes were constructed for all isolates using bcftools (v1.9)^18^. A multiple sequence alignment was constructed using the consensus genomes, and phylogenetic reconstruction was performed using iqtree software (v1.6.12, -m GTR + G)^19^.

## Results

**Figure 1:**
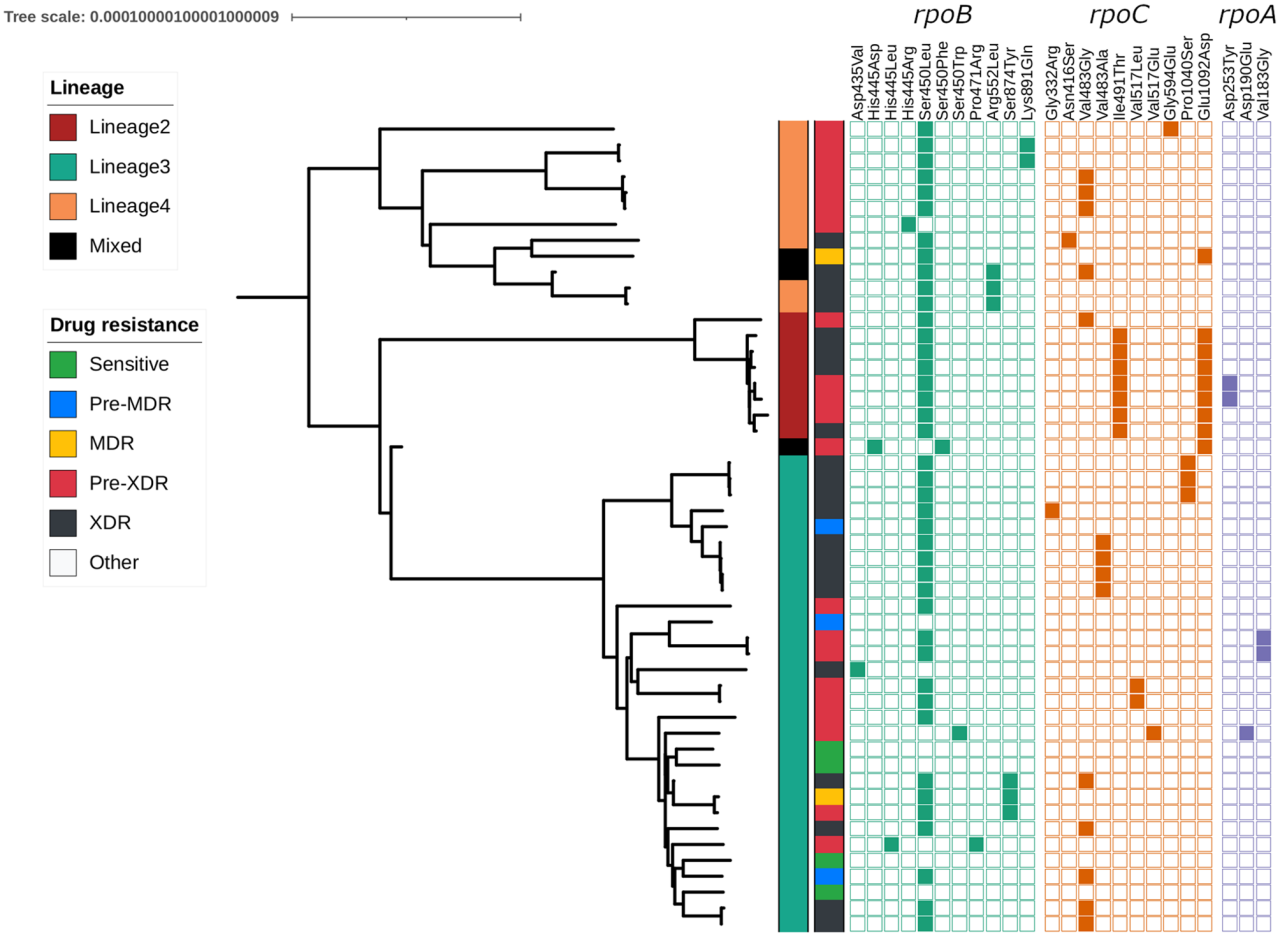
Phylogenetic tree of *M. tuberculosis* strains (n = 51), with their lineages, multi-drug resistance profiles and specific mutations in *rpoB, rpoA,* and *rpoC*. The phylogenetic tree was created using a maximum likelihood approach. The first vertical band to the right of the tree denotes the lineage. The second vertical band denotes the drug resistance phenotype. The squares show the presence of a specific mutation.

High quality data was generated for fifty-one isolates with a median of 5,186,316 read pairs per isolate. Trimming removed low-quality reads leading to the retention of on average 70.9% of pairs with both reads and 97.2% with at least one read. Classification of the reads using Kraken software reported high percentages classified as Mycobacterium (median: 99.7%). Mapping of the reads led to an average depth of 216-fold coverage, and 99% of the genome with at least tenfold coverage. Of the fifty-one *M. tuberculosis* isolates, the majority were from lineage 3 (30, 58.8%), and were sourced from female patients (n = 29, 56.9%) and previously treated cases (n = 30, 58.8%) (Table 1). We identified four mixed infections, consisting of different sub-lineages (4.8 and 2.2.1, 4.9 and 3, 3 and 2.2.1, 3 and 4.6.2.1) (Table 1; Supplementary Table S1; Fig. 1), potentially reflecting the high transmission setting. By comparing the allelic frequencies at each mutation position with the predicted frequency of the strains within a mixture, we assigned mutations to the individual strains wherever possible. The phenotypic and genotypic drug resistance profiles were concordant across all samples at the dominant strain level, but the genomic data revealed the complexity of the four mixed lineage infections. In particular, one sample contained a combination of MDR-TB and isoniazid-resistant strains, whilst the other three isolate samples were combinations of MDR-TB with MDR-TB (n = 2) or pan susceptible (n = 1) strains (Supplementary Table S1).

**Table 1:** Patient demographic and genotypic data of the 51 *M. tuberculosis* isolates.

Characteristic	#	%
**Patient treatment history**		
Never treated	3	5.9
On treatment	11	21.6
Previously treated	30	58.8
Unknown	7	13.7
**Patient gender**		
Female	29	56.9
Male	22	43.1
**Collection time point**		
Diagnosis	18	35.3
Follow up	33	64.7
***M. tuberculosis* lineage**^**a**^		
2	8	15.7
3	30	58.8
4	10	19.6
Mixed lineage	3	5.9
**Phenotypic/genotypic DST**		
Susceptible	4	7.8
Rifampicin resistant	2	3.9
Isoniazid resistant	1	2.0
MDR-TB^b^	44	86.3

*DST* drug susceptibility test, *MDR-TB* multidrug-resistant TB.

^a^There are five mixed infections, with three mixed lineage and two same lineage infections (see Supplementary Table S1).

^b^MDR-TB or MDR-TB+, with 5 mixed infections involving MDR-TB (see Supplementary Table S1).

Isolates with isoniazid resistance (n = 45) presented with underlying haplotypes *katG* S315T (31/45), *fabG1* − 15C > T + *katG* S315T (6/45), *fabG1* − 15C > T (3/45), *fabG1* − 15C > T + *ahpC* − 54C > T (3/45), *fabG1* − 15C > T + *katG* D311G (1/45), and *katG* V1A (1/45) (Supplementary Table S1). The RR-TB and MDR-TB isolates (n = 46) all had established rifampicin related mutations: *rpoB* S450L (41/46), R552 (3/46), H445D/L/R (3/46), S874Y (3/46), and S450W/F (2/46) (Table 2). We found two potential new mutations in *rpoB* (K891Q, P471R) (Table 2), but they appeared on the same background as established resistance markers (*rpoB* H445L and S450L) (Table 3; Supplementary Table S1). Of the RR-TB and MDR-TB samples (n = 46), 34 (73.9%) had at least one potential compensatory mutation, the majority in *rpoC* (32/46) and a minority in *rpoA* (5/46), including three isolates with mutations in both genes. The most common *rpoC* mutations were V483G (10), E1092D (9), I491T (7), V483A (4), P1040S (3), and K891Q (2), and when observed previously, have been present in predominantly RR-TB strains (Table 2), thereby reflecting their potential compensatory role. The *rpoA* mutations included D253Y (2/46), V183G (2/46), and D190E (1/46), all appearing to be previously unreported (Table 2), but only V183G was present on the background without *rpoC* mutations (Table 3). Interestingly, the only potentially unreported *rpoC* mutation (V517E) was in a mixed infection (MDR-TB/isoniazid-resistant) with *rpoB* S450W and *rpoA* D190E present (Table 3, Supplementary Table S1).

**Table 2:** Non-synonymous mutations in rifampicin candidate genes of *M. tuberculosis.*

Gene	Nucleotide	Mutation	Freq	Global # (% RR-TB)^a^	P/NP	HP/HB
*rpoB*	761155	S450L	41	5395 (99)	P-NP	HP-HB
*rpoC*	764817	V483G	10	473 (98)	NP-NP	HB-HB
*rpoC*	766645	E1092D	9	1462 (78)	P-P	HP-HP
*rpoC*	764,841	I491T	7	125 (100)	NP-P	HB-HP
*rpoC*	764817	V483A	4	154 (99)	NP-NP	HB-HB
*rpoB*	761461	R552L	3	27 (100)	P-NP	HP-HB
*rpoB*	762427	S874Y	3	14 (100)	P-P	HP-HP
*rpoC*	766487	P1040S	3	79 (98)	NP-P	HB-HP
***rpoB***	**762477**	K891Q	2	0 (–)	P-P	HP-HP
*rpoC*	764918	V517L	2	55 (100)	NP-NP	HB-HB
***rpoA***	**3877751**	D253Y#	2	0 (–)	P-P	HP-HP
***rpoA***	**3877960**	V183G	2	0 (–)	NP-NP	HB-HB
*rpoB*	761110	D435V	1	627 (99)	P-NP	HP-HB
*rpoB*	761139	H445D	1	322 (100)	P-P	HP-HP
*rpoB*	761140	H445L	1	106 (96)	P-NP	HP-HB
*rpoB*	761140	H445R	1	107 (96)	P-P	HP-HP
*rpoB*	761155	S450W	1	142 (96)	P-NP	HP-HB
***rpoB***	**761218**	P471R	1	0 (–)	NP-P	HB-HP
*rpoC*	764363	G332R	1	59 (100)	NP-P	HB-HP
*rpoC*	764,616	N416S	1	30 (100)	P-P	HP-HP
***rpoC***	**764919**	V517E	1	0 (–)	NP-P	HB-HP
*rpoC*	765150	G594E	1	4582 (27)	NP-P	HB-HP
***rpoA***	**3877938**	D190E	1	0 (–)	P-P	HP-HP
*rpoB*	761155/761156	S450F	1	46 (100)	P-NP	HP-HB

Bolded are unreported by Napier et al. (~ 35 k).

*RR-TB* rifampicin-resistant TB, *P* polar, *NP* non-polar, *HP* hydrophilic, *HB* hydrophobic.

^a^From Napier et al. (~ 35 k).

**Table 3:** Combinations of *rpoB, rpoC*, and *rpoA* mutations present in the 51 *M. tuberculosis* isolates.

*rpoB* mutations	*rpoC* mutations	*rpoA* mutations	# isolates
S450L	V483G	–	8
S450L	I491T, E1092D	–	5
–	–	–	5^a^
S450L	V483A	–	4
S450L	–	–	3
S450L	P1040S	–	3
S450L	V517L	–	2
S450L, K891Q	–	–	2
S450L	I491T, E1092D	D253Y	2
S450L, S874Y	–	–	2
S450L	–	V183G	2
S450L	G594E	–	1
S450L	N416S	–	1
S450L	G332R	–	1
S450L, R552L	–	–	1
H445L, P471R	–	–	1
D435V	–	–	1
S450L, S874Y (0.67)	V483G (0.41)	–	1
S450L, R552L (0.88)	–	–	1
S450W (0.42)	V517E (0.44)	D190E (0.54)	1
S450L (0.21)	E1092D (0.20)	–	1^b^
H445R (0.89)	–	–	1^b^
H445D (0.34), S450F (0.70)	E1092D (0.48)	–	1^b^
S450L, R552L (0.66)	V483G (0.27)	–	1^b^

– Absent, () proportion of reads in a mixed infection and < 1.

^a^Rifampicin susceptible.

^b^Mixed infections.

**Figure 2:**
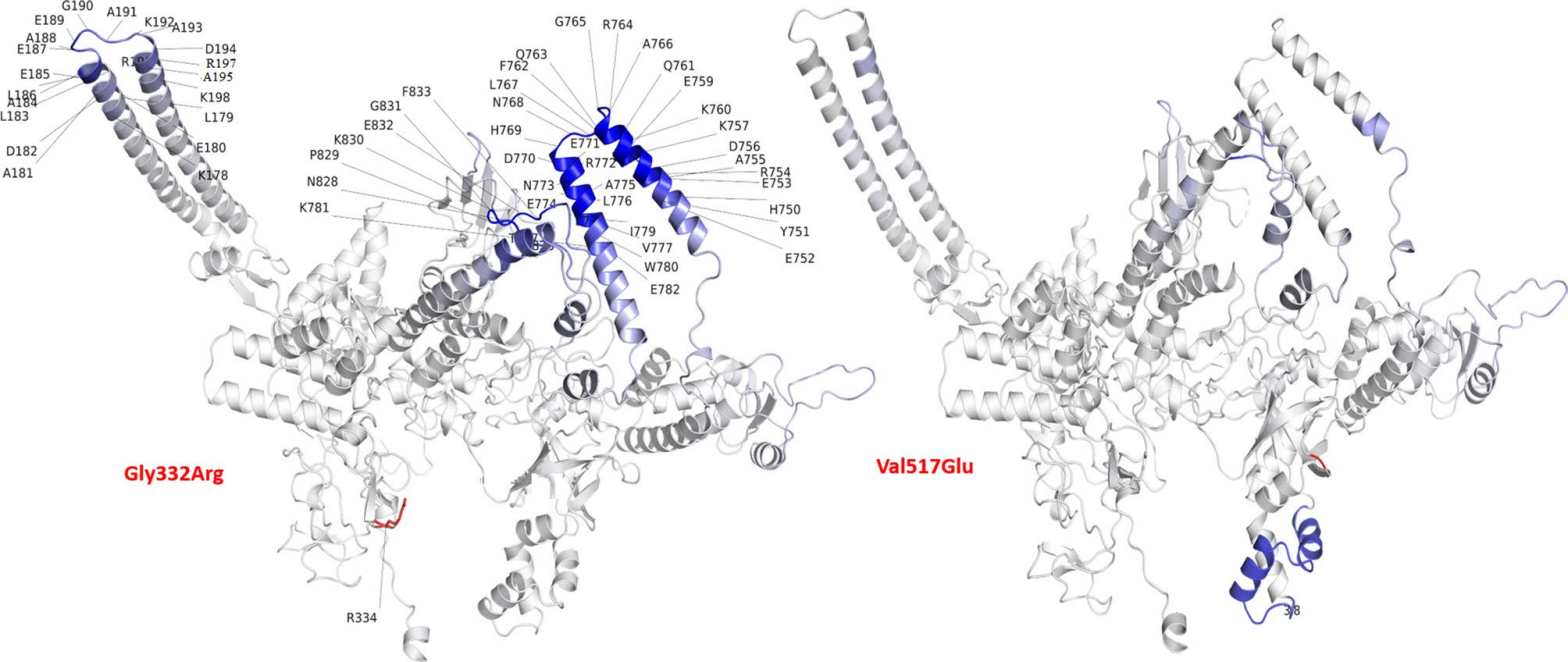
Effect of the *rpoC* G332R and V517E point mutations on RpoC protein dynamics. ΔΔG; Free energy difference. ΔΔS_Vib_ ENCoM; vibrational entropy energy. The effects have been predicted through the DynaMut online server. The increasing (red) and decreasing (blue) molecular flexibility effects of point mutations have been depicted. The total energy calculated for mutations shows a stabilizing effect on protein structure.

**Figure 3:**
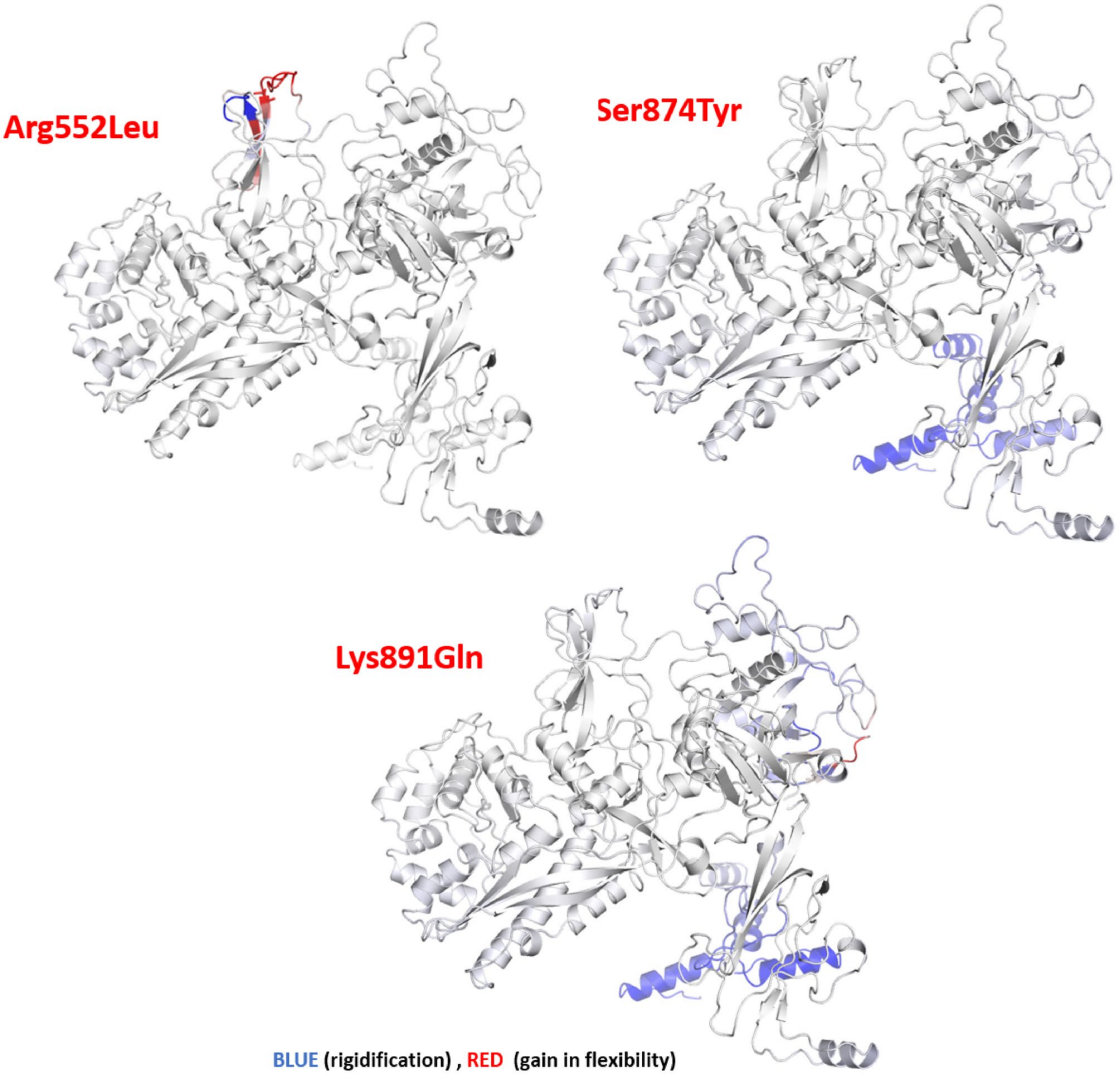
Effects of R552L, S874Y, and K891Q mutations on the RpoB protein structure. ΔΔG; Free energy difference. ΔΔS_Vib_ ENCoM; vibrational entropy energy. The increasing (red) and decreasing (blue) molecular flexibility effects of point mutations have been depicted.

**Figure 4:**
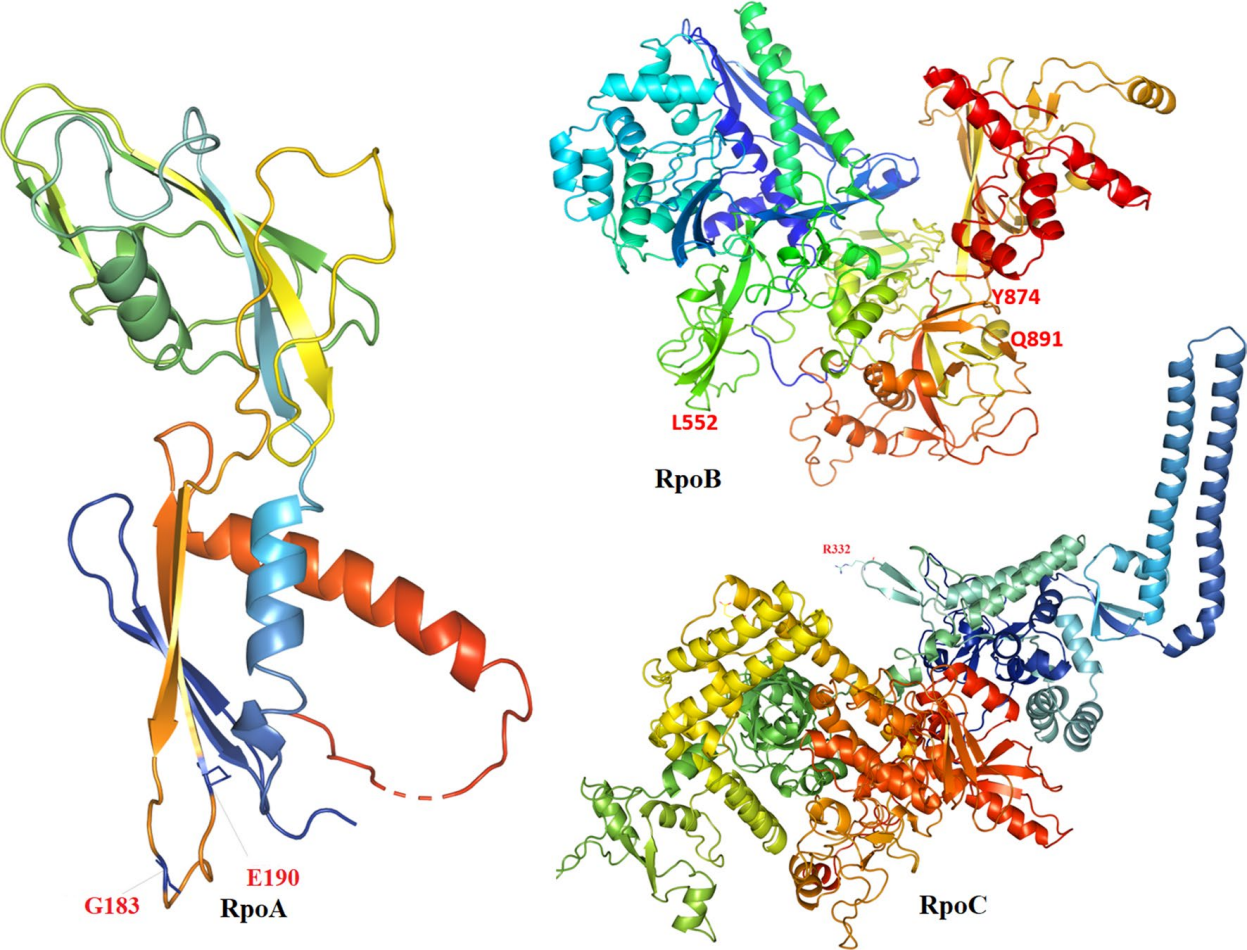
Location of residues in RpoA, RpoB, RpoC proteins.

We assessed in silico the impact of eight low frequency mutations (*rpoB* P471R, R552L, S874Y, K891Q; *rpoA* V183G, D190E; *rpoC* G332, V517E) on the stability and flexibility of appropriate protein structures (RpoA, RpoB, RpoC) (Table 4; Figs. 2, 3, 4). These mutations all have low frequency in our study (1–3 isolates) and in the 35 k dataset (< 30 isolates). Note, the impact of the potentially unreported *rpoA* D253Y mutation was not assessed, as residues of D253 for RpoA could not be modelled because of the eight mutations considered, all but two (*rpoB *R552L; *rpoA* D190E) had a potentially stabilizing effect. The *rpoC* G332R mutation had the highest stability and lowest flexibility effects (Table 4) and is found exclusively in RR-TB strains (Table 2). The *rpoA* D190E led to the least stable and highest flexibility effects. This mutation was present on the uncommon *rpoB* S450W and *rpoC* V517E mutation background, where each mutation is found almost exclusively in RR-TB strains (Table 2).

**Table 4:** Protein structure stability and flexibility effects for low frequency mutations.

Gene	Mutation^a^	Freq	P/NP	HP/HB	Energy^b^	Flexibility^c^
*rpoB*	R552L	3	P-NP	HP-HB	− 0.123	− 0.008
*rpoB*	S874Y	3	P-P	HP-HP	0.776	− 0.188
*rpoB*	**K891Q**	2	P-P	HP-HP	0.270	− 0.397
*rpoA*	V183G	2	NP-NP	HB-HB	0.063	− 0.908
*rpoB*	**P471R**	1	NP-P	HB-HP	0.682	− 0.443
*rpoC*	G332R	1	NP-P	HB-HP	1.196	− 3.975
*rpoC*	**V517E**	1	NP-P	HB-HP	0.120	− 0.162
*rpoA*	**D190E**	1	P-P	HP-HP	− 0.500	0.104

Low frequency mutations from Table 2, where the number of samples with that mutation in the 35 k dataset was < 300.

^a^Mutation bolded if potentially unreported; *P* polar, *NP* non-polar, *HP* hydrophilic, *HB* hydrophobic.

^b^Total energy (ΔΔG: kcal/mol), where +ve values are stabilizing and −ve values are destabilizing.

^c^ΔΔSVib ENCoM (kcal/mol-1.K-1), where negative values reflect decreasing molecule flexibility; note, residues of D253 have not been modelled in PDB structure (PDB ID: 6c04).

## Discussion

Resistance to rifampicin (RR-TB) has been associated with mutations in an 81 bp hot-spot region of the *rpoB* gene. However, mutations in *rpoA* and *rpoC*, have also been linked to RR-TB, particularly in a compensatory role^20^. We identified 24 mutations across *rpoA, rpoB* and rpoC *genes* in 51 *M. tuberculosis* (46 RR-TB) isolates, including six mutations [*rpoA* (2), *rpoB* (3), and *rpoC* (1)], which were not found in a large collection (~ 35 k) of global clinical isolates. All RR-TB could be explained by known mutations, including the *rpoB* S450L (41/46 isolates), which is the most prevalent mutation globally (~ 64%)^20^ and in Pakistan (~ 74%^21^; ~ 71%^22^). Four *rpoB* mutations (R552L, S874Y, K891Q, P471R) appear not to have been reported by recent genetic studies from Khyber Pakhtunkhwa^21–23^. Some recently reported *rpoB* mutations in Pakistan (L430P, H445P/Y, L452P^21^; S450Q, P454H, G455D^22^; D435G^21,22^, D435Y^21,23^), which are of much lower frequency than the S450L mutation and sometimes reported in combination with it, were not present in our RR-TB isolates. Similarly, all isoniazid resistance could be explained by known mutations, including the *katG* S315T (37/45), which has a high frequency globally (~ 75%) and in Pakistan (> 89%^21,23^). The majority of RR-TB/MDR-TB isolates had mutations in *rpoC* (32/46), which is a locus known to restore the fitness of rifampicin-resistant bacteria carrying mutations in *rpoB*^24^. Other studies have found a lower prevalence of *rpoC* mutations in MDR isolates (< 30%)^6,25,26^, including a study in Pakistan^26^, and inconsistencies may be due to differences in sampling sites and strategies. Eight mutations in *rpoC* (G332R, N416S, V483A, V517E/L, G594E, P1040S, E1092D) appear not to have been reported in recent genetic studies from Khyber Pakhtunkhwa^21–23^. The evidence for a prominent compensatory role of *rpoA* in RR-TB is less strong. Our study identified only three *rpoA* mutations, of which two appear not to have been reported before, and were in the presence of *rpoC* mutations. In general, potential *rpoA* and *rpoC* compensatory mutations and mechanisms need to be validated experimentally in fitness studies, or through large-scale studies involving phylogenetic analysis of longitudinally collected strains.

Phenotypic DST is considered to be the gold standard method to infer resistance. However, some studies have reported that liquid culture systems (e.g. the MGIT platform) often fail to detect low-levels of RR-TB^27,28^, including those involving H445D/L/R mutations^27,29^, which have been linked to treatment failure or relapse^30–32^. The role of such mutations can be investigated using protein structural analysis^33^, and previous studies have shown that mutations may affect the structural dynamics of proteins, making them weak targets, resulting in drug resistance^4,34,35^. Our in silico analysis revealed that the *rpoC* G332R mutation, which is rare (< 0.05%) globally and exclusively in RR-TB isolates, led to high protein stability and less flexibility, which may be favorable features for compensatory effects. This mutation has been previously reported in a South African setting^5^, where it was observed to originate through homoplastic evolution and thus likely to confer a selective advantage. Several of the other low-frequency mutations in the three genes have been reported previously (*rpoB* R552L^36^, *rpoB* S874Y^37^ and *rpoA* V183G^6^) in different settings and have been proposed as potential compensatory mutations. For other low-frequency mutations including *rpoB* K891Q, *rpoB* P471R, *rpoC* V517E, *rpoA* D190E and *rpoA* D253Y, we did not find any previous reports in the literature. These mutations fall outside the rifampicin resistance determining regions and co-occur with known resistance mutations, potentially to compensate for the fitness impact of the resistance mutations.

A potential limitation of the phenotypic DST exposed by our genomic analysis is its robustness to mixed infections and heteroresistance. Only the WHO recommended concentration of the rifampicin drug was assessed, and dominant rifampicin resistance mutants within MDR-TB mixes can complicate the phenotypic interpretation of any low-level rifampicin resistance mutants. In our study, five instances of within-host resistance mechanism diversity were observed as the result of mixed infections. Three instances involved mixed-lineage infections, two of which were a mixture of MDR-TB strains and one was a mixture of a pan-susceptible and an MDR-TB strain. Interestingly, the MDR-TB mixtures contained different resistance-conferring mutations in *rpoB, katG* and the *inhA* promoter region, as well as likely compensatory mutations in *rpoC* and potentially *rpoA*. These mutations were found in the same proportions within the raw data as lineage variants, indicating the different strains carry resistance markers. Heteroresistance was also observed in isolates with a single lineage. This observation could be indicative of acquired resistance, where mutations have not become fixed in the within-host bacterial population. Almost all single-lineage RR-TB isolates contained the *rpoB* S450L at a completely fixed level in the sequenced population. Some of these strains possessed additional mutations in *rpoB, rpoC* and *rpoA* at unfixed proportions (e.g. *rpoB* R552L, *rpoB* S874Y, *rpoC* V483G and *rpoA* D190E). Based on this observation it is likely that these mutations were acquired after the *rpoB* S450L, which confers high-level resistance and serve as compensatory mutations to restore the fitness penalty of the resistance mutation.

The application of sequencing technologies in TB endemic regions will assist clinical management and personalised anti-TB drug approaches, as well as disease control through surveillance activities. The management of MDR-TB strains is essential for the control of TB. In our study, we found that the mutations underlying MDR-TB are established variants. Known and putative drug resistance markers in established loci are detectable using low-cost sequencing-based approaches (e.g. amplicon-based), suitable for a low resource setting. These methods are also robust to mixed infections and heteroresistance^35,38^, which were present in our study and likely driven by the high transmission setting. Large-scale approaches using whole genome sequencing and analysis will assist with understanding the epidemiology and risk groups or factors underpinning the transmission of TB, as well as provide insights into drug resistance and compensatory mechanisms. Such insights will inform the deployment of anti-TB drug regimens and disease control tools and strategies in high burden settings, such as Pakistan.

## Conclusion

Whole genome sequencing for TB clinical management and disease control will have the greatest benefit in complex community outbreaks in endemic regions. Our study in the Khyber Pakhtunkhwa province in Pakistan has provided a characterization of circulating rifampicin resistance and MDR-TB mutations. Such insights will assist sequencing-informed diagnosis for proactive TB patient management, and the deployment of anti-TB drug regimens and surveillance activities.

## Supplementary Information

Supplementary Table S1.

## Data Availability

The raw sequence data is available from the EBI ENA (Accession number PRJEB43284).
