# Author Correction: Characterization of rifampicin-resistant *Mycobacterium tuberculosis* in Khyber Pakhtunkhwa, Pakistan

**DOI:** 10.1038/s41598-021-94687-3

**Published:** 2021-07-19

**Authors:** Anwar Sheed Khan, Jody E. Phelan, Muhammad Tahir Khan, Sajid Ali, Muhammad Qasim, Gary Napier, Susana Campino, Sajjad Ahmad, Otavio Cabral-Marques, Shulin Zhang, Hazir Rahman, Dong-Qing Wei, Taane G. Clark, Taj Ali Khan

**Affiliations:** 1 Department of Microbiology, Kohat University of Science and Technology, Kohat, Pakistan; 2 Provincial Tuberculosis Reference, Laboratory Hayatabad Medical Complex, Peshawar, Pakistan; 3 Faculty of Infectious and Tropical Diseases, London School of Hygiene & Tropical Medicine, London, UK; 4 Institute of Molecular Biology and Biotechnology (IMBB), The University of Lahore. KM, Defense Road, Lahore, 58810 Pakistan; 5 Institute of Basic Medical Science, Khyber Medical University, Peshawar, KP Pakistan; 6 Department of Immunology, Institute of Biomedical Sciences, University of Sao Paulo, São Paulo, SP Brazil; 7 Department of Clinical and Toxicological Analyses, School of Pharmaceutical Sciences, University of São Paulo, São Paulo, SP Brazil; 8 Network of Immunity in Infection, Malignancy, and Autoimmunity (NIIMA), Universal Scientific Education and Research Network (USERN), São Paulo, Brazil; 9 Department of Microbiology and Immunology, Shanghai Jiao Tong University School of Medicine, Shanghai, China; 10 Department of Microbiology, Abdul Wali Khan University, Mardan, Pakistan; 11 State Key Laboratory of Microbial Metabolism, School of Life Sciences and Biotechnology, and Joint Laboratory of International Cooperation in Metabolic and Developmental Sciences, Ministry of Education, Shanghai Jiao Tong University, 800 Dongchuan Road, Minhang District, Shanghai, 200240 China; 12 Faculty of Epidemiology and Population Health, London School of Hygiene & Tropical Medicine, London, UK

Correction to: *Scientific Reports* 10.1038/s41598-021-93501-4, published online 09 July 2021

The original version of this Article contained an error in the spelling of the author Otavio Cabral-Marques which was incorrectly given as Otavio Marques Cabral. The original Article has been corrected.

