# Supplementary material for: Characterization of rifampicin-resistant Mycobacterium tuberculosis in Khyber Pakhtunkhwa, Pakistan
Source: Sci Rep. 2021 Jul 9;11:14194. doi: 10.1038/s41598-021-93501-4 (PMC8270973; doi:10.1038/s41598-021-93501-4)
Supplement: Supplementary file 1 — Supplementary Table S1. [file 41598_2021_93501_MOESM1_ESM.docx]

**S1 Table. Multidrug-resistant mutations**

| Isolate  ID | lineage | DR Phenotype | Isoniazid mutations | *rpoB* | *rpoC* | *rpoA* |
| --- | --- | --- | --- | --- | --- | --- |
| 1 | 2.2.1 | MDR-TB | *katG* S315T | S450L | I491T, E1092D | - |
| 2 | 3 | RR-TB | - | S450L | V483G | - |
| 3 | 3 | Sens. | - | - | - | - |
| 5 | 3 | MDR-TB | *katG* S315T | S450L, S874Y (0.67) | V483G (0.41) | - |
| 6 | 3 | MDR-TB | *katG* S315T | S450L | - | - |
| 7 | 3 | Sens. | - | - | - | - |
| 9 | 3 | MDR-TB | *fabG1* -15C>T, *katG* S315T | S450L | V483G | - |
| 10 | 4.9 | MDR-TB | *katG* S315T | S450L, R552L (0.88) | - | - |
| 11 | 3 | MDR-TB | *katG* S315T | S450L, S874Y | - | - |
| 12 | 3 | MDR-TB | *katG* S315T | S450L | V483A | - |
| 13 | 3 | MDR-TB | *katG* S315T | S450L | V483G | - |
| 14 | 4.9 | MDR-TB | *katG* S315T | S450L, R552L | - | - |
| 15 | 3 | MDR-TB | *katG* S315T | S450L | G332R | - |
| 16* | 4.9/3 | MDR/MDR | *fabG1* -15C>T (0.26), *katG* S315T | S450L, R552L (0.66) | V483G (0.27) | - |
| 18 | 2.2.1 | MDR-TB | *katG* S315T | S450L | I491T, E1092D | - |
| 19 | 2.2.1 | MDR-TB | *katG* S315T | S450L | I491T, E1092D | - |
| 20 | 3 | MDR-TB | *katG* S315T | S450L | V483A | - |
| 21 | 3 | MDR-TB | *fabG1* -15C>T | S450L | P1040S | - |
| 22 | 3 | RR-TB | - | S450L | - | - |
| 23 | 3 | Sens. | - | - | - | - |
| 24 | 3 | MDR-TB | *katG* S315T | S450L | V483A | - |
| 28 | 3 | MDR-TB | *katG* S315T | S450L | V483A | - |
| 30* | 4.8/2.2.1 | MDR/Sens. | *katG* S315T (0.21) | S450L (0.21) | E1092D (0.20) | - |
| 31 | 3 | MDR-TB | *katG* S315T | D435V | - | - |
| 32 | 3 | Sens | - | - | - | - |
| 35 | 3.1.2.1 | MDR-TB | *katG* S315T | S450L | - | V183G |
| 36* | 3/4.6.2.1 | MDR/Iso | *katG* S315T | H445R (0.89) | - | - |
| 38 | 3 | MDR-TB | *fabG1* -15C>T | S450L | P1040S | - |
| 44 | 3 | MDR-TB | *fabG1* -15C>T, *katG* S315T | S450L | V483G | - |
| 45 | 3 | MDR-TB | *fabG1* -15C>T | S450L | P1040S | - |
| 46 | 3 | MDR-TB | *fabG1* -15C>T, *katG* S315T | H445L, P471R | - | - |
| 47 | 2.2.1 | MDR-TB | *katG* S315T | S450L | I491T, E1092D | - |
| 48 | 4.8 | MDR-TB | f*abG1* -15C>T, *katG* S315T | S450L | N416S | - |
| 49 | 4.5 | MDR-TB | *fabG1* -15C>T, *ahpC* -54C>T | S450L | V483G | - |
| 52 | 3.1.2.1 | Iso. | *katG* S315T | - | - | - |
| 55 | 3 | MDR-TB | *katG* S315T | S450L | V517L | - |
| 56 | 2.2.1 | MDR-TB | *katG* S315T | S450L | V483G | - |
| 57 | 3 | MDR-TB | *katG* S315T | S450L, S874Y | - | - |
| 58 | 3.1.2.1 | MDR-TB | *katG* S315T | S450L | - | V183G |
| 59 | 2.2.1 | MDR-TB | *katG* S315T | S450L | I491T, E1092D | D253Y |
| 60 | 3 | MDR-TB | *katG* S315T | S450L | - | - |
| 63 | 4.5 | MDR-TB | *fabG1* -15C>T, *ahpC* -54C>T | S450L | V483G | - |
| 64 | 3 | MDR/Iso | *katG* V1A | S450W (0.42) | V517E (0.44) | D190E (0.54) |
| 65 | 2.2.1 | MDR-TB | *katG* S315T | S450L | I491T, E1092D | - |
| 66 | 2.2.1 | MDR-TB | *katG* S315T | S450L | I491T, E1092D | D253Y |
| 68* | 3/2.2.1 | MDR/MDR | *fabG1* -15C>T (0.65), *katG* S315T (0.39) | H445D (0.34), S450F (0.70) | E1092D (0.48) | - |
| 69 | 3 | MDR-TB | *katG* S315T | S450L | V517L | - |
| 70 | 4.5 | MDR-TB | *katG* S315T | S450L, K891Q | - | - |
| 71 | 4.1.1 | MDR-TB | *fabG1* -15C>T, *katG* D311G | S450L | G594E | - |
| 72 | 4.5 | MDR-TB | *katG* S315T | S450L, K891Q | - | - |
| 73 | 4.5 | MDR-TB | *fabG1* -15C>T, *ahpC* -54C>T | S450L | V483G | - |

* mixed infections; () proportion of reads in a mixed infection and <1; DR = drug resistance; Sens. = pan susceptible; Iso = Isoniazid; MDR-TB = multidrug resistant; RR-TB = rifampicin resistant
